# Clinical, Psychological, and Behavioral Factors Associated with Fatigue in Multiple Sclerosis

**DOI:** 10.3390/jcm15218314

**Published:** 2026-10-26

**Authors:** Wioletta Mikuľáková, Eleonóra Klímová, Miloslav Gajdoš, Štefánia Andraščíková, Joanna Rosińczuk, Lucia Demjanovič Kendrová

**Affiliations:** 1 Department of Physiotherapy, Faculty of Health Care, University of Prešov in Prešov, Partizánska 1 St., 080 01 Prešov, Slovakia; eleonora.klimova@unipo.sk (E.K.); miloslav.gajdos@unipo.sk (M.G.); lucia.kendrova@unipo.sk (L.D.K.); 2 Department of Midwifery, Faculty of Health Care, University of Prešov in Prešov, Partizánska 1 St., 080 01 Prešov, Slovakia; stefania.andrascikova@unipo.sk; 3 Department of Nursing, Faculty of Nursing and Midwifery, Wroclaw Medical University, Bartla 5, 51-618 Wrocław, Poland; joanna.rosinczuk@umw.edu.pl or joanna.rosinczuk@unipo.sk; 4 Department of Nursing, Faculty of Health Care, University of Prešov in Prešov, Partizánska 1 St., 080 01 Prešov, Slovakia

**Keywords:** multiple sclerosis, fatigue, depression, pain, physical activity, disability, kinesiophobia

## Abstract

**Background/Objectives:** Fatigue is one of the most common and disabling symptoms of multiple sclerosis (MS) and is associated with clinical, psychological, and behavioral factors. This study aimed to identify factors independently associated with fatigue severity in patients with MS and assess their relative contribution to fatigue variability. **Methods:** This cross-sectional study included 148 patients with MS in Slovakia (105 women and 43 men; mean age 42.21 ± 9.84 years). Neurological disability and fatigue were assessed using the Expanded Disability Status Scale (EDSS) and Modified Fatigue Impact Scale (MFIS), respectively. Pain was assessed using the SF-36 Bodily Pain domain. Depressive symptoms, anxiety, kinesiophobia, and physical activity were assessed using the Zung Self-Rating Depression Scale (SDS), Beck Anxiety Inventory (BAI), Tampa Scale for Kinesiophobia (TSK), and Godin Leisure-Time Exercise Questionnaire (GLTEQ), respectively. Hierarchical linear regression was used to identify factors independently associated with fatigue. **Results:** The final model explained 50.7% of MFIS variance (R^2^ = 0.507; adjusted R^2^ = 0.463). Lower SF-36 Bodily Pain scores, indicating greater pain and pain-related interference (β = −0.412; *p* < 0.001), more severe depressive symptoms (SDS; β = 0.266; *p* < 0.001), and lower physical activity (GLTEQ; β = −0.151; *p* = 0.027), were independently associated with greater fatigue. The association between EDSS and fatigue was not statistically significant after adjustment (*p* = 0.162). SDS, BAI, and GLTEQ jointly increased the explained variance by 9.1 percentage points (ΔR^2^ = 0.091; *p* < 0.001). Kinesiophobia did not explain statistically significant additional variance (ΔR^2^ = 0.001; *p* = 0.539). The estimated indirect association between depressive symptoms and fatigue through kinesiophobia was not statistically significant (B = −0.010; 95% bootstrap CI −0.063 to 0.025). **Conclusions:** Fatigue in MS is multifactorial. Pain, depressive symptoms, and physical activity were independently associated with fatigue. These findings suggest the relevance of considering these factors in multidisciplinary fatigue assessment in MS. Evidence of an additional independent association involving kinesiophobia was insufficient, but a clinically relevant association cannot be excluded.

## 1. Introduction

Multiple sclerosis (MS) is a chronic, immune-mediated disease of the central nervous system (CNS), in which inflammatory and neurodegenerative processes contribute to demyelination, axonal damage, and neuronal loss [1]. The multifocal and heterogeneous nature of CNS involvement results in substantial variability in disease course, neurological impairment, and functional consequences among people with MS [2]. In addition to neurological deficits directly related to disease activity and the accumulation of disability, people with MS frequently experience symptoms whose severity cannot be fully explained by neurological impairment alone [3].

Fatigue is among the most common and disabling symptoms of multiple sclerosis, with prevalence estimates ranging from approximately 60% to 80% across studies [4,5,6]. It is characterized by a pronounced lack of physical and/or mental energy perceived by the patient as interfering with usual or desired activities [4]. Fatigue has a substantial impact on quality of life and, for many patients, represents one of the most disabling symptoms of the disease [7]. It is also associated with important socioeconomic consequences, particularly reduced work capacity, shorter working hours, or loss of employment [8,9].

Despite its high prevalence and considerable clinical impact, fatigue in MS remains a complex and insufficiently understood symptom. Its pathophysiology is multifactorial and involves interactions among neurobiological, clinical, psychological, and behavioral mechanisms [10]. Both primary mechanisms related to the disease itself, particularly inflammatory and neurodegenerative processes, and secondary factors arising from comorbid conditions and the cumulative burden of disease may contribute to the onset, severity, and persistence of fatigue. These factors include neurological disability, MS disease course, pain, treatment-related iatrogenic factors, depression and other psychological factors, sleep disturbances, and reduced levels of physical activity [10,11,12].

Neurological disability and disease course are among the clinical factors significantly associated with fatigue [13,14,15]. Higher scores on the Expanded Disability Status Scale (EDSS) are associated with greater fatigue, and more severe fatigue is more frequently observed in patients with progressive forms of MS. However, this relationship may be partly influenced by the greater level of disability that characterizes progressive forms of the disease [10].

Pain is another clinical factor relevant to the assessment of fatigue in MS. Pain-related sleep disruption and the sedating effects of some medications used to manage pain may contribute to fatigue [12]. Accounting for pain may therefore help clarify the independent associations of psychological and behavioral factors with fatigue.

Psychological factors, particularly depression, also play an important role. Depressive symptoms may clinically overlap with fatigue, for example through reduced motivation and anhedonia, which may make it difficult to distinguish depression from fatigue directly related to MS [12]. Several studies have demonstrated significant associations between depressive symptoms and fatigue severity [16,17,18,19].

The relationship between psychological factors and fatigue is likely complex and may involve both biological and behavioral mechanisms. Depressive and anxiety symptoms may be accompanied by reduced motivation, sleep disturbances, and limitations in physical activity, which may subsequently contribute to physical deconditioning and the persistence or worsening of fatigue [10].

Several cross-sectional studies have demonstrated an inverse relationship between physical activity levels and fatigue in people with MS, while meta-analyses have also indicated the moderate effect of exercise interventions on fatigue reduction [20,21]. The relationship between fatigue and physical activity may be bidirectional, and maladaptive behavioral responses may also contribute to the persistence of fatigue [22,23]. One potential behavioral factor associated with persistent fatigue is kinesiophobia, defined as an irrational, debilitating, and intense fear of movement and physical activity arising from a perceived vulnerability to reinjury or worsening of the condition [24]. Although kinesiophobia is increasingly being investigated in neurological and rheumatological conditions, its role in the context of MS-related fatigue remains insufficiently understood. Kese et al. [25] demonstrated significant relationships among fatigue-related kinesiophobia, fatigue, and depressive symptoms in patients with MS, suggesting a possible role of fear-avoidance responses in fatigue. These findings indicate a potential behavioral link between psychological symptoms and fatigue in people with MS.

Avoidance behavior related to fear of movement may contribute to reduced physical activity and subsequent deconditioning, potentially creating a vicious cycle of physical inactivity and symptom persistence [26,27].

Current evidence suggests that fatigue in MS should be understood within a biopsychosocial framework integrating neurological, psychological, and behavioral factors. Although the associations of fatigue with neurological disability and psychological symptoms in MS are well documented, it remains unclear whether kinesiophobia provides additional explanatory information beyond these factors. Assessing its contribution while simultaneously accounting for pain, depressive and anxiety symptoms, and physical activity is particularly important. This approach allows the unadjusted association between kinesiophobia and fatigue to be distinguished from its unique contribution within a comprehensive model. Therefore, this study aimed to identify factors independently associated with fatigue in people with MS and specifically assess the incremental contribution of kinesiophobia using hierarchical linear regression analysis. We hypothesized that greater neurological disability, greater pain severity, more severe depressive and anxiety symptoms, greater kinesiophobia, and lower levels of physical activity would be associated with greater fatigue.

## 2. Materials and Methods

### 2.1. Study Design and Participants

This cross-sectional study was conducted at the Multiple Sclerosis Diagnosis and Treatment Centre of the J. A. Reiman University Hospital in Prešov between 2021 and 2024. The study was conducted in accordance with the Declaration of Helsinki, and the protocol was approved by the Ethics Committee of the Faculty of Health Care, University of Prešov in Prešov (No. 052020) on 6 November 2020. Before inclusion in the study, all participants were informed about its aims and procedures and provided written informed consent to participate. Participation in the study was voluntary and was not financially compensated. This study is reported in accordance with the Strengthening the Reporting of Observational Studies in Epidemiology (STROBE) statement for cross-sectional studies [28].

All patients who met the inclusion criteria and attended an outpatient examination during the study period were consecutively enrolled. In total, 205 patients with a confirmed diagnosis of multiple sclerosis were assessed for eligibility. After applying the predefined inclusion and exclusion criteria, 148 patients were included in the final analysis.

The study included adult patients with a diagnosis of multiple sclerosis (MS) established according to the revised McDonald criteria [29,30,31] or, for patients diagnosed before their introduction, the Poser criteria [32]. Additional inclusion criteria were age ≥ 18 years, a stable clinical condition without relapse during the three months preceding study enrollment, an Expanded Disability Status Scale (EDSS) score between 1 and 6, a Mini-Mental State Examination (MMSE) score of at least 23 points, and signed informed consent. The EDSS range of 1–6 was selected to reduce difficulties in interpreting physical activity and kinesiophobia in patients with substantial motor impairment. In these patients, low physical activity may primarily reflect a limited capacity for movement, making it difficult to distinguish physical limitations from activity restriction due to fear of movement. The same EDSS range has been used in previous research examining kinesiophobia and physical activity in patients with MS [24].

Patients without a confirmed diagnosis of multiple sclerosis, those with another neurological disorder, individuals with insufficient cognitive abilities to ensure reliable cooperation, patients who had experienced a relapse within the three months preceding assessment, individuals younger than 18 years, and those who declined participation were excluded. In total, 57 patients were excluded from the analysis: 41 because of an EDSS score > 6, 3 because of another neurological disorder, 5 because of insufficient cognitive function, 5 because of relapse during the three months preceding assessment, and 3 because they were younger than 18 years.

### 2.2. Minimum Sample Size Estimation

The minimum sample size was initially estimated using the recommendation of Khamis and Kepler for multiple regression models (*n* = 20 + 5k). Applying this recommendation to the revised model with 12 predictor terms yielded a minimum sample size of 80 participants. A sensitivity analysis for the overall regression model indicated that a sample of 148 participants, with 12 predictor terms and α = 0.05, could detect an effect size of approximately Cohen’s f^2^ = 0.126 with 80% statistical power. For a medium effect size (f^2^ = 0.15), the corresponding statistical power was approximately 87.7% [33].

### 2.3. Measurements

A cross-sectional design was employed to collect clinical and questionnaire-based data from individuals with multiple sclerosis (MS) living in Slovakia. Clinical information, including disability status, disease duration, clinical course, and immunomodulatory treatment, was obtained from medical records with the assistance of the treating physician. For the present analysis, treatment status was recorded as a binary variable (receiving vs. not receiving immunomodulatory therapy), without distinguishing between individual disease-modifying therapies or treatment regimens. Neurological status was assessed by neurologists experienced in the diagnosis and management of MS, and disability was quantified using the Expanded Disability Status Scale (EDSS).

Questionnaire data were collected during a scheduled follow-up visit conducted within one week of the clinical assessment. Participants completed the study measures during face-to-face interviews with a member of the research team. Sociodemographic characteristics, including sex, educational attainment, and employment status, were recorded using a structured questionnaire.

Patient-reported outcomes were evaluated using established and validated instruments. Fatigue was assessed with the Modified Fatigue Impact Scale (MFIS), while fear of movement was measured using the Tampa Scale for Kinesiophobia (TSK). Physical activity was evaluated with the Godin Leisure-Time Exercise Questionnaire (GLTEQ). Pain intensity and pain-related interference were assessed using the SF-36 Bodily Pain domain. Depressive symptoms were evaluated using the Zung Self-Rating Depression Scale (SDS), whereas anxiety symptoms were assessed with the Beck Anxiety Inventory (BAI).

#### 2.3.1. Expanded Disability Status Scale (EDSS)

The Expanded Disability Status Scale (EDSS) ranges from 0 to 10 in 0.5-point increments and is used to quantify neurological disability in people with multiple sclerosis. The neurologist first evaluates the degree of impairment in the eight functional systems of the CNS and compares the score obtained with the standards for patient mobility, for example the ability to walk a certain distance. The combination of results in functional systems and walking ability determines the overall EDSS score—the rate of functional incapacity and disability [34].

#### 2.3.2. Modified Fatigue Impact Scale (MFIS)

The Slovak version of the MFIS was used to assess fatigue. The scale evaluates the impact of fatigue on physical, cognitive, and psychosocial functioning. Items are rated from 0 (no problem) to 4 (extreme problem) according to the participant’s experience during the previous month. The physical subscale contains 9 items (0–36 points), the cognitive subscale 10 items (0–40 points), and the psychosocial subscale 2 items (0–8 points). The total MFIS score is 0–84 points [14]. A higher score indicates a greater impact of fatigue on daily functioning across physical, cognitive, and psychosocial domains. The MFIS total score was analyzed as a continuous variable in correlation and regression analyses. The commonly used clinical threshold of MFIS ≥38 was used to identify patients with clinically significant fatigue for descriptive purposes and group comparisons [13].

#### 2.3.3. Tampa Scale for Kinesiophobia (TSK)

The Slovak version of the Tampa Scale for Kinesiophobia (TSK) consists of 17 items aimed at assessing the subjective degree of fear of movement. Responses are recorded on a four-point Likert scale from “strongly disagree” to “strongly agree”. Each item is scored from 1 to 4, with items 4, 8, 12, and 16 reverse-scored. The total score ranges from 17 to 68 points. A total score of 17 represents the minimum possible score, with higher scores indicating greater kinesiophobia. Scores >37 have commonly been used to indicate a high level of kinesiophobia, with higher scores reflecting greater kinesiophobia [35].

#### 2.3.4. Godin Leisure-Time Exercise Questionnaire (GLTEQ)

The GLTEQ was used to assess physical activity level. It includes three questions assessing the frequency of strenuous (e.g., running), moderate (e.g., brisk walking), and light (e.g., easy walking) physical activity performed for more than 15 min during a typical week. Weekly frequencies are multiplied by weighting factors of 9 for strenuous, 5 for moderate, and 3 for light activity. The total leisure-time activity score is calculated as follows: (9 × strenuous) + (5 × moderate) + (3 × light) [36].

#### 2.3.5. Zung Self-Rating Depression Scale (SDS)

The Slovak version of the standardized SDS was used to assess depression. It contains 20 items, each rated from 1 (never or rarely) to 4 (most or all of the time). A raw total score of 20–80 is converted into a 100-point SDS index (a raw total score × 1.25) [37].

#### 2.3.6. Beck Anxiety Inventory (BAI)

Anxiety was assessed using the Slovak version of the Beck Anxiety Inventory (BAI). BAI is a self-assessment inventory with 21 items to measure the intensity of anxiety. Participants rate the extent to which each symptom bothered them during the previous week on a 4-point scale [38].

#### 2.3.7. The SF-36 Bodily Pain Domain (SF-36 BP)

The SF-36 Bodily Pain domain assesses pain intensity and pain-related interference. Previous psychometric research has supported its internal consistency and convergence with other pain measures. The SF-36 composite Bodily Pain score was derived from items assessing pain intensity and interference over the past 4 weeks following published guidelines [39]; lower scores reflect more severe and limiting pain.

#### 2.3.8. Mini-Mental State Examination (MMSE)

The MMSE is a widely used 30-point screening instrument assessing several domains of cognitive functioning [40].

### 2.4. Statistical Analysis

Statistical analysis was performed on a sample of 148 patients with multiple sclerosis. There were no missing data for variables included in the primary correlation and regression analyses; therefore, all 148 participants were included in these analyses. Continuous variables were summarized using the mean and standard deviation (SD) and median (Me). For group comparisons, data were presented as the median, first and third quartiles (Q1–Q3), and interquartile range (IQR). Categorical variables were presented as absolute and relative frequencies.

For the descriptive characterization of clinically significant fatigue, patients were divided according to the total Modified Fatigue Impact Scale (MFIS) score into a group without clinically significant fatigue (MFIS < 38) and a group with clinically significant fatigue (MFIS ≥ 38) [13]. Differences in continuous variables between these groups were assessed using the Mann–Whitney U test. Effect size was expressed as the coefficient *r*, calculated as |Z|/√N. Values of *r* of approximately 0.10, 0.30, and 0.50 were interpreted as small, medium, and large effects, respectively.

Associations between the MFIS total score and age, disease duration, neurological disability (EDSS), depressive symptoms (SDS), anxiety (BAI), pain (SF-36 BP), kinesiophobia (TSK), and physical activity level (GLTEQ) were assessed using Spearman’s correlation coefficient (*ρ*).

To identify factors independently associated with fatigue, hierarchical linear regression analysis was subsequently performed, with the MFIS total score as the dependent variable. Variables were entered into the model in four blocks based on their clinical and theoretical relevance. In the first step, demographic variables (age and sex) were entered. In the second step, clinical variables (disease duration, EDSS, immunomodulatory therapy, MS clinical phenotype, and pain [SF-36 Bodily Pain score]) were added. In the third step, depressive symptoms (SDS), anxiety (BAI), and physical activity level (GLTEQ) were entered. In the final step, kinesiophobia (TSK) was added to assess its additional contribution to explaining the variance in fatigue beyond the other variables. MS clinical phenotype was entered using two indicator variables, with relapsing–remitting MS (RRMS) as the reference category.

For each regression model, R^2^, adjusted R^2^, ΔR^2^, and the statistical significance of the change in R^2^ were reported. For individual predictors, unstandardized regression coefficients (B), standard errors (SE), standardized regression coefficients (β), 95% confidence intervals, *t* values, and *p* values were reported. The assumptions of linear regression were assessed by examining linearity, normality and homoscedasticity of residuals, and independence of errors. Multicollinearity was assessed using tolerance and the variance inflation factor (VIF). The unique contributions of SDS, BAI, and GLTEQ were assessed by omitting each predictor from the final model and calculating the decrease in R^2^ and corresponding partial R^2^.

The final model was evaluated using residual Q–Q and residual-versus-fitted plots, the Shapiro–Wilk test, and the studentized Breusch–Pagan test. A supplementary RESET test assessed the contribution of squared and cubed fitted values. Influence diagnostics included externally studentized residuals, leverage, Cook’s distance, and GLTEQ DFBETAS. Sensitivity analyses used HC3 standard errors with t-based inference, model refitting after excluding observations with Cook’s distance > 4/n, leave-one-out refitting, and 10,000 case-bootstrap samples with percentile 95% confidence intervals. All complete eligible observations were retained in the primary analysis; influence thresholds were used for screening only.

An exploratory mediation analysis was performed to estimate the indirect association between depressive symptoms and fatigue through kinesiophobia. SDS score was entered as the independent variable, TSK score as the mediator, and MFIS total score as the dependent variable. Both the mediator and outcome models were adjusted for age, sex, disease duration, EDSS, immunomodulatory therapy, MS clinical phenotype, pain (SF-36 BP), anxiety (BAI), and physical activity (GLTEQ). The significance of the indirect effect was evaluated using bootstrap confidence intervals based on 10,000 resamples. An indirect effect was considered statistically significant when the 95% bootstrap confidence interval did not include zero. All statistical analyses were performed using MedCalc^®^ Statistical Software, version 23, and IBM SPSS Statistics, version 30. Statistical significance was set at *p* < 0.05.

## 3. Results

The study sample consisted of 148 patients with a confirmed diagnosis of multiple sclerosis, including 105 women (70.95%) and 43 men (29.05%). The mean age of the participants was 42.21 ± 9.84 years, with an age range of 21–71 years. The mean disease duration was 10.14 ± 6.70 years (range, 1–37 years). Regarding the clinical course of the disease, relapsing–remitting multiple sclerosis was the most common form (*n* = 107; 72.30%), followed by secondary progressive (*n* = 29; 19.59%) and primary progressive multiple sclerosis (*n* = 12; 8.11%). The mean EDSS score was 3.73 ± 1.52, with a median of 4.00. Regarding educational attainment, most patients had completed secondary education (*n* = 84; 56.76%), followed by higher education (*n* = 62; 41.89%), while two participants (1.35%) had completed primary education. A total of 46 patients (31.08%) had no work capacity restrictions, 19 patients (12.84%) reported restricted work capacity, and 83 patients (56.08%) were receiving a disability pension. At the time of assessment, 92 patients (62.16%) were receiving immunomodulatory therapy, whereas 56 patients (37.84%) were not. A high level of kinesiophobia (TSK > 37) was observed in 98 patients (66.22%), whereas 50 patients (33.78%) had a low level of kinesiophobia (TSK ≤ 37). Detailed sociodemographic, clinical, and patient-reported characteristics of the study sample are presented in Table 1.

Patients with clinically significant fatigue (MFIS ≥ 38; *n* = 79) were significantly older, had a longer disease duration, a higher level of neurological disability as assessed by the EDSS, had significantly lower SF-36 BP scores and higher depressive symptom scores (SDS) compared with patients without clinically significant fatigue (MFIS < 38; *n* = 69). The fatigue group also showed a significantly lower level of physical activity as assessed by the GLTEQ. A large effect size was observed for pain (SF-36 BP; *r* = 0.585), whereas medium effect sizes were observed for neurological disability (EDSS; *r* = 0.442), depressive symptoms (SDS; *r* = 0.417), and disease duration (*r* = 0.311). Age (*r* = 0.288) and physical activity level (GLTEQ; *r* = 0.210) showed small effect sizes. Differences in anxiety (BAI; *p* = 0.132) and kinesiophobia (TSK; *p* = 0.098) were not statistically significant (Table 2).

Spearman’s correlation analysis showed that higher total MFIS scores were significantly associated with older age (ρ = 0.254; *p* = 0.002), longer disease duration (ρ = 0.226; *p* = 0.006), greater neurological disability as assessed by the EDSS (ρ = 0.412; *p* < 0.001), and more severe depressive symptoms (ρ = 0.462; *p* < 0.001). Lower SF-36 BP scores were significantly associated with higher MFIS total scores (ρ = −0.624; *p* < 0.001). In contrast, higher levels of physical activity were associated with lower fatigue severity (ρ = −0.325; *p* < 0.001). Correlations of fatigue with anxiety (BAI; ρ = 0.150; *p* = 0.069) and kinesiophobia (TSK; ρ = 0.127; *p* = 0.123) were not statistically significant (Table 3).

Hierarchical linear regression analysis was performed to identify factors independently associated with fatigue severity in patients with MS. In the first step, demographic variables (age and sex) were entered into the model and explained 6.2% of the variance in the total MFIS score (R^2^ = 0.062; *p* = 0.010). After the addition of clinical variables (disease duration, EDSS, immunomodulatory therapy, MS clinical phenotype, and pain [SF-36 BP]), the explained variance significantly increased to 41.5% (ΔR^2^ = 0.353; *p* < 0.001).

In the third step, psychological and behavioral variables (depressive symptoms—SDS, anxiety—BAI, and physical activity level—GLTEQ) were added to the model, resulting in a further significant increase in the explained variance to 50.6% (ΔR^2^ = 0.091; *p* < 0.001). This increase represents the joint contribution of SDS, BAI, and GLTEQ, rather than the contribution of any individual variable. After kinesiophobia (TSK) was added in the fourth step, the coefficient of determination increased to 50.7% (R^2^ = 0.507; adjusted R^2^ = 0.463); however, the additional 0.14 percentage points of explained variance were not statistically significant (ΔR^2^ = 0.001; *p* = 0.539). Changes in R^2^ were calculated from unrounded values.

In the final model, lower SF-36 BP scores, indicating greater pain and pain-related interference (β = −0.412; *p* < 0.001), more severe depressive symptoms as assessed by the SDS (β = 0.266; *p* < 0.001), and lower physical activity levels as assessed by the GLTEQ (β = −0.151; *p* = 0.027) were independently associated with greater fatigue. Age, sex, disease duration, EDSS, immunomodulatory therapy, MS clinical phenotype, anxiety (BAI), and kinesiophobia (TSK) were not statistically significant after adjustment for the other variables. Multicollinearity diagnostics did not indicate substantial collinearity among the independent variables, with all tolerance values > 0.37 and all variance inflation factor (VIF) values < 2.65 (Table 4).

In the final model, the unique contributions of SDS, BAI, and GLTEQ corresponded to decreases in R^2^ of 0.0465, 0.0001, and 0.0183, respectively, when each variable was omitted while retaining all other predictors. The corresponding partial R^2^ values were 0.0862 for SDS, 0.0002 for BAI, and 0.0358 for GLTEQ.

Exploratory covariate-adjusted mediation analysis did not provide statistically significant evidence of an indirect association between depressive symptoms and fatigue through kinesiophobia. Covariates included age, sex, disease duration, EDSS, immunomodulatory therapy, MS clinical phenotype, pain (SF-36 BP), anxiety (BAI), and physical activity (GLTEQ). The estimated indirect association of SDS with MFIS through TSK was B = −0.010, and its 95% percentile bootstrap confidence interval included zero (95% CI: −0.063 to 0.025; 10,000 resamples). The direct association between depressive symptoms and fatigue remained significant after inclusion of TSK and the covariates (B = 0.499; 95% CI: 0.222 to 0.775; *p* < 0.001). These cross-sectional estimates do not establish temporal ordering or causal mediation.

In the 148-participant model, residual diagnostics did not identify marked departures from normality (Shapiro–Wilk *p* = 0.151), evidence of heteroscedasticity (studentized Breusch–Pagan *p* = 0.171), or evidence against the fitted functional form in the supplementary RESET test (*p* = 0.767). Ten observations exceeded Cook’s distance 4/n, and one externally studentized residual exceeded 3 in absolute value. The GLTEQ association was inverse in the primary model (B = −0.162; 95% CI −0.305 to −0.019; *p* = 0.027), with HC3 inference (95% CI −0.310 to −0.013; *p* = 0.033), after excluding observations with Cook’s distance values > 4/n (B = −0.198; *p* = 0.002), and in the case bootstrap (95% CI −0.308 to −0.029). All leave-one-out coefficients remained negative, although *p*-values ranged from 0.012 to 0.062 (145/148 below 0.05).

## 4. Discussion

The main finding of this study is that fatigue severity in patients with multiple sclerosis is associated with a combination of clinical, psychological, and behavioral factors. The final model explained 50.7% of the variance in fatigue, with pain, depressive symptoms, and physical activity level independently associated with fatigue. Demographic variables explained only a small proportion of the variance in fatigue, whereas the addition of clinical variables significantly improved the proportion of explained variance. The inclusion of psychological factors and physical activity increased the explained variance of the model by a further 9.1 percentage points.

In our study, no statistically significant independent association between neurological disability assessed using the EDSS and fatigue severity was observed after adjustment for the other variables, including pain. However, the lack of statistical significance does not exclude a clinically relevant association. This finding differs from those of several studies reporting an association between greater neurological disability and greater fatigue in patients with MS [15]. In contrast to our findings, the longitudinal analysis by Ghajarzadeh et al. [41] demonstrated that greater physical disability significantly increased the risk of developing persistent fatigue. Patients with lower levels of disability remained free from clinically significant fatigue for approximately eight months longer. Morrison and Stuifbergen [42] found that functional limitations were the strongest predictor of both the physical and psychosocial dimensions of fatigue in patients with long-standing multiple sclerosis. Tarasiuk et al. [19] also reported that the risk of both fatigue and depression increases proportionally with increasing disability as assessed by the EDSS. The restricted range of neurological disability in our sample (EDSS 1–6) may also have limited our ability to detect an association between disability and fatigue.

Our findings indicate an independent association between pain and fatigue and support the multifactorial nature of fatigue in MS. Lower SF-36 BP scores, indicating greater pain and pain-related interference with daily activities, were associated with greater fatigue severity after adjustment for the other variables. Kratz et al. [43] assessed pain, fatigue, depressed mood, and physical activity concurrently in daily life, illustrating the value of examining these symptoms within a broader clinical and behavioral context.

Adding depressive symptoms, anxiety, and physical activity to the model containing demographic and clinical variables, including pain, increased the explained variance in fatigue from 41.5% to 50.6%. This increase represents the joint contribution of these three variables. In the final model, pain, depressive symptoms, and physical activity were independently associated with fatigue. These findings suggest the relevance of considering these factors in a comprehensive assessment of fatigue in patients with MS.

Among the psychological variables examined, depressive symptoms showed the strongest association with fatigue. Even after controlling for age, sex, disease duration, neurological disability, pain, physical activity level, and the other variables, depressive symptoms remained independently associated with higher MFIS scores. This finding is consistent with several studies demonstrating a close relationship between depression and fatigue in multiple sclerosis [16,17,18,19,44]. Fidao et al. [45] highlighted the important role of depressive symptoms in the relationship between fatigue and poorer mental health-related quality of life in patients with multiple sclerosis.

The close relationship between depression and fatigue is also supported by Tarasiuk et al. [19], who highlighted their frequent co-occurrence and partially overlapping symptomatology. The authors also noted that the relationship between depression and fatigue may be bidirectional: depressive symptoms may contribute to the development and severity of fatigue, whereas persistent fatigue may subsequently increase the risk of depressive symptoms.

In contrast to depressive symptoms, anxiety did not show a statistically significant independent association with fatigue after adjustment for the other variables. This finding does not exclude a clinically relevant association between anxiety and fatigue. In contrast, the longitudinal study by Ghajarzadeh et al. [41] identified anxiety as a risk factor for the future development of fatigue. The difference between their findings and ours may be related to differences in study design, the instruments used to assess anxiety and fatigue, and the statistical approach. While our study assessed factors associated with current fatigue severity in a cross-sectional sample, Ghajarzadeh et al. [41] examined the development of fatigue longitudinally.

Our study demonstrated that lower physical activity levels were independently associated with greater fatigue. This finding is consistent with previous evidence showing that people with MS who experience fatigue have lower levels of physical activity, take fewer steps, and spend more time in sedentary behavior compared with those without fatigue [21,46]. Fatigue may also represent an important barrier to physical activity, as greater fatigue has been associated with lower adherence to home-based exercise programs in patients with MS [47]. However, the relationship between physical activity and fatigue may be bidirectional. Greater fatigue may limit daily physical activity, whereas long-term physical inactivity may contribute to persistent fatigue through deconditioning, reduced muscle strength, and decreased functional capacity. This may result in a mutually reinforcing cycle of fatigue and physical inactivity [43,48,49].

The importance of physical activity is further supported by studies showing that exercise interventions, including aerobic and resistance training, may reduce fatigue and improve functional status in patients with MS [50,51,52]. Our results suggest the relevance of considering physical activity in a comprehensive assessment of fatigue in MS. The inverse association between physical activity and fatigue was supported by several sensitivity analyses, although its statistical significance was sensitive to individual observations. This association should therefore be considered modest and interpreted cautiously. The cross-sectional design does not permit conclusions about the causality of the observed association or the effectiveness of interventions aimed at increasing physical activity to reduce fatigue.

Kinesiophobia did not show a statistically significant independent association with fatigue after adjustment for the other variables. However, a clinically relevant association cannot be excluded, particularly given the modest sample size and the potential influence of movement limitations on kinesiophobia scores. The findings also do not exclude a possible indirect relationship with fatigue through other behavioral mechanisms, particularly those related to physical activity, as fear of movement may promote avoidance behavior and activity restriction. Our findings also did not provide statistically significant evidence of an indirect association between depressive symptoms and fatigue through kinesiophobia. These cross-sectional estimates do not establish temporal ordering or causal mediation and should not be interpreted as evidence that kinesiophobia has no role in the association between depressive symptoms and fatigue.

The contribution of this study lies in evaluating whether kinesiophobia provides additional explanatory information about fatigue beyond demographic and clinical characteristics, pain, depressive and anxiety symptoms, and physical activity. By distinguishing the unadjusted association from the incremental contribution of kinesiophobia, this analysis helps clarify its role within a broader biopsychosocial model of MS-related fatigue.

### Strengths and Limitations

This study has several limitations that should be considered when interpreting the findings. First, the cross-sectional design does not allow causal relationships to be established or the direction of associations between fatigue, depressive symptoms, physical activity, and the other variables to be clearly determined. Therefore, the results of the mediation analysis should be interpreted as statistical associations rather than evidence of a temporal or causal mechanism.

Several variables, including fatigue, depressive and anxiety symptoms, pain, kinesiophobia, and physical activity, were assessed using self-report questionnaires and may therefore have been influenced by subjective perception and response bias. In particular, overlapping somatic and psychological content in the SDS and MFIS, together with common-method bias arising from self-reported assessment, may have influenced the magnitude of the association between depressive symptoms and fatigue.

Another limitation is the inclusion of patients with EDSS scores ranging from 1 to 6, which may limit the generalizability of the findings to individuals with greater neurological disability. However, this criterion was selected because of the nature of the behavioral factors assessed, particularly physical activity and kinesiophobia. Assessment of physical activity using the GLTEQ is based on the frequency of physical activities of different intensities, and in patients with severely restricted mobility, the ability to perform such activities would be substantially limited by the degree of disability itself. Similarly, interpreting kinesiophobia in patients with very severe motor impairment or wheelchair dependence would be problematic because movement restriction might primarily result from neurological deficits rather than fear of movement. Restricting the EDSS range therefore allowed the relationships of physical activity and kinesiophobia with fatigue to be assessed in a population in which independent physical activity remained realistically feasible. Nevertheless, the findings cannot be directly extrapolated to patients with advanced disability. The exclusion of 41 patients with EDSS scores > 6 may also have introduced selection bias and altered the distribution of fatigue severity in the sample.

Immunomodulatory therapy was analyzed as a dichotomous variable according to its presence or absence. This approach was chosen because the aim of the study was not to compare the effects of individual treatment regimens on fatigue but to assess the relative importance of clinical, psychological, and behavioral factors. Moreover, dividing patients according to individual treatment types would have resulted in small and uneven subgroups, potentially reducing the stability of estimates in the multivariable model. This binary classification did not allow potential differences between high-efficacy and platform therapies, including treatment-related differences in fatigue, to be examined. Although MS clinical phenotype was included in the regression models, the small progressive subgroups, particularly PPMS (*n* = 12), limited the precision of phenotype-specific estimates. Sleep disturbances, medication-related fatigue, and relevant comorbidities were not systematically assessed and could not be included in the regression models. These unmeasured factors may have influenced the observed associations, and residual confounding cannot be excluded. Despite these limitations, the study simultaneously assessed clinical, psychological, and behavioral factors and enabled their independent relationships with fatigue to be evaluated within a single regression model.

## 5. Conclusions

The findings of this study highlight the multifactorial nature of fatigue in patients with multiple sclerosis. Greater pain and pain-related interference, more severe depressive symptoms, and lower physical activity levels were associated with greater fatigue after adjustment for the other variables included in the model, suggesting the relevance of considering these factors in comprehensive multidisciplinary fatigue assessment.

Evidence of an additional explanatory contribution of kinesiophobia or an indirect association between depressive symptoms and fatigue through kinesiophobia was insufficient; however, a clinically relevant association cannot be excluded.

The cross-sectional design does not establish causal relationships or the effectiveness of interventions targeting these factors. Future research should include longitudinal evaluation, objective physical activity monitoring, systematic assessment of sleep, symptomatic medication use and comorbidities, and trials of integrated psychological and rehabilitation interventions.

## Figures and Tables

**Table 1:** Sociodemographic, clinical, and patient-reported characteristics of the study population. (N = 148).

Characteristic	*n*	%	Mean ± SD/Me
Age (years)			42.21 ± 9.84/42.00
Gender—Men	43	29.05	
Gender—Women	105	70.95	
Education—Primary	2	1.35	
Education—High school	84	56.76	
Education—Higher education	62	41.89	
Work capacity—Without restrictions	46	31.08	
Work capacity—With restrictions	19	12.84	
Work capacity—Disability pension	83	56.08	
Course of the disease—Relapsing–remitting	107	72.30	
Course of the disease—Primary progressive	12	8.11	
Course of the disease—Secondary progressive	29	19.59	
Immunomodulatory therapy—Yes	92	62.16	
Immunomodulatory therapy—No	56	37.84	
Disease duration (years)			10.14 ± 6.70/9.00
EDSS score			3.73 ± 1.52/4.00
Fatigue (MFIS total score)			38.65 ± 17.38/39.00
Depression (SDS)			52.39 ± 9.28/52.50
Anxiety (BAI)			18.91 ± 9.58/18.00
Pain (SF-36 BP)			59.13 ± 22.11/55.00
Kinesiophobia (TSK)			39.27 ± 6.73/39.00
Kinesiophobia (TSK ≤ 37)	50	33.78	
Kinesiophobia (TSK > 37)	98	66.22	
Physical activity (GLTEQ)			17.86 ± 16.19/17.00

Abbreviations: BAI, Beck Anxiety Inventory; EDSS, Expanded Disability Status Scale; GLTEQ, Godin Leisure-Time Exercise Questionnaire; Me, median; MFIS, Modified Fatigue Impact Scale; *n*, number of patients; SD, standard deviation; SDS, Zung Self-Rating Depression Scale; SF-36 BP, SF-36 Bodily Pain score; TSK, Tampa Scale for Kinesiophobia.

**Table 2:** Comparison of clinical characteristics between patients with and without clinically significant fatigue.

Variable	No Fatigue (*n* = 69)			Fatigue Present (*n* = 79)			Mann–Whitney U Test		
	Median	Q1–Q3	IQR	Median	Q1–Q3	IQR	Z	*p*	Effect Size (*r*)
Age	40.00	35.00–45.00	10.00	44.00	39.50–51.00	11.50	−3.507	<0.001	0.288
Disease duration	7.00	5.00–10.00	5.00	11.00	7.00–15.00	8.00	−3.782	<0.001	0.311
EDSS	3.00	2.00–4.00	2.00	4.00	3.50–5.50	2.00	−5.376	<0.001	0.442
SDS	47.50	41.25–56.25	15.00	56.00	51.25–62.50	11.25	−5.068	<0.001	0.417
BAI	16.00	10.00–23.00	13.00	19.00	14.00–28.00	14.00	−1.507	0.132	0.124
SF-36 BP	77.50	57.50–87.50	30.00	45.00	32.50–55.00	22.50	7.113	<0.001	0.585
TSK	39.00	34.00–44.00	10.00	41.00	36.00–45.50	9.50	−1.653	0.098	0.136
GLTEQ	17.00	9.00–23.00	14.00	12.00	6.00–18.50	12.50	2.560	0.010	0.210

Abbreviations: EDSS = Expanded Disability Status Scale; SDS = Zung Self-Rating Depression Scale; BAI = Beck Anxiety Inventory; SF-36 BP = SF-36 Bodily Pain score; TSK = Tampa Scale for Kinesiophobia; GLTEQ = Godin Leisure-Time Exercise Questionnaire; Q1–Q3 = first to third quartile; IQR = interquartile range; *r* = effect size.

**Table 3:** Spearman correlations between MFIS total score and study variables.

Variable	MFIS (ρ)	*p*
Age	0.254	0.002
Disease duration	0.226	0.006
EDSS	0.412	<0.001
SDS	0.462	<0.001
BAI	0.150	0.069
SF-36 BP	−0.624	<0.001
TSK	0.127	0.123
GLTEQ	−0.325	<0.001

Abbreviations: MFIS = Modified Fatigue Impact Scale; EDSS = Expanded Disability Status Scale; SDS = Zung Self-Rating Depression Scale; BAI = Beck Anxiety Inventory; SF-36 BP = SF-36 Bodily Pain score; TSK = Tampa Scale for Kinesiophobia; GLTEQ = Godin Leisure-Time Exercise Questionnaire; ρ = Spearman’s rank correlation coefficient.

**Table 4:** Hierarchical linear regression analysis of factors associated with MFIS total score.

Independent Variable	B	SE	β	95% CI	t	*p*	Tolerance	VIF
**Model 1: R^2^ = 0.062, Adjusted R^2^ = 0.049, ΔR^2^ = 0.062, *p* for ΔR^2^ = 0.010**								
Constant	26.934	7.612	–	11.888 to 41.979	3.538	**<0.001**	–	–
Age	0.428	0.143	0.242	0.145 to 0.711	2.984	**0.003**	0.982	1.019
Sex	−3.715	3.098	−0.097	−9.838 to 2.409	−1.199	0.232	0.982	1.019
**Model 2: R^2^ = 0.415, Adjusted R^2^ = 0.381, ΔR^2^ = 0.353, *p* for ΔR^2^ < 0.001**								
Constant	53.348	8.624	–	36.296 to 70.400	6.186	**<0.001**	–	–
Age	0.021	0.149	0.012	−0.274 to 0.315	0.138	0.890	0.591	1.692
Sex	−0.397	2.630	−0.010	−5.598 to 4.804	−0.151	0.880	0.886	1.128
Duration	−0.244	0.237	−0.094	−0.712 to 0.225	−1.027	0.306	0.503	1.987
EDSS	2.876	1.134	0.252	0.633 to 5.118	2.535	**0.012**	0.427	2.342
Immunomodulatory therapy	2.513	2.680	0.070	−2.785 to 7.812	0.938	0.350	0.748	1.336
Pain (SF-36 BP)	−0.420	0.057	−0.534	−0.533 to −0.307	−7.339	**<0.001**	0.794	1.259
PPMS vs. RRMS	−0.140	4.475	−0.002	−8.988 to 8.709	−0.031	0.975	0.847	1.180
SPMS vs. RRMS	0.824	3.356	0.019	−5.811 to 7.460	0.246	0.806	0.712	1.404
**Model 3: R^2^ = 0.506, Adjusted R^2^ = 0.466, ΔR^2^ = 0.091, *p* for ΔR^2^ < 0.001**								
Constant	30.109	11.553	–	7.262 to 52.956	2.606	**0.010**	–	–
Age	0.067	0.139	0.038	−0.208 to 0.342	0.482	0.631	0.587	1.702
Sex	−2.784	2.497	−0.073	−7.722 to 2.153	−1.115	0.267	0.849	1.178
Duration	−0.082	0.223	−0.032	−0.524 to 0.359	−0.370	0.712	0.490	2.039
EDSS	1.499	1.113	0.131	−0.701 to 3.699	1.347	0.180	0.383	2.612
Immunomodulatory therapy	2.306	2.522	0.065	−2.682 to 7.293	0.914	0.362	0.729	1.371
Pain (SF-36 BP)	−0.333	0.057	−0.423	−0.445 to −0.220	−5.853	**<0.001**	0.696	1.437
PPMS vs. RRMS	−1.858	4.188	−0.029	−10.140 to 6.423	−0.444	0.658	0.835	1.198
SPMS vs. RRMS	2.031	3.164	0.047	−4.227 to 8.288	0.642	0.522	0.691	1.446
SDS	0.489	0.138	0.261	0.215 to 0.763	3.530	**<0.001**	0.665	1.503
BAI	0.053	0.120	0.029	−0.184 to 0.290	0.443	0.658	0.831	1.203
GLTEQ	−0.163	0.072	−0.152	−0.305 to −0.020	−2.259	**0.026**	0.807	1.239
**Model 4: R^2^ = 0.507, Adjusted R^2^ = 0.463, ΔR^2^ = 0.001, *p* for ΔR^2^ = 0.539**								
Constant	24.868	14.372	–	−3.556 to 53.292	1.730	0.086	–	–
Age	0.064	0.139	0.036	−0.212 to 0.339	0.456	0.649	0.587	1.705
Sex	−2.686	2.508	−0.070	−7.645 to 2.274	−1.071	0.286	0.845	1.183
Duration	−0.081	0.224	−0.031	−0.523 to 0.361	−0.362	0.718	0.490	2.039
EDSS	1.576	1.122	0.138	−0.643 to 3.796	1.405	0.162	0.378	2.646
Immunomodulatory therapy	2.510	2.549	0.070	−2.532 to 7.552	0.985	0.327	0.717	1.395
Pain (SF-36 BP)	−0.324	0.059	−0.412	−0.440 to −0.208	−5.526	**<0.001**	0.657	1.523
PPMS vs. RRMS	−1.739	4.202	−0.027	−10.049 to 6.571	−0.414	0.680	0.833	1.200
SPMS vs. RRMS	2.215	3.186	0.051	−4.085 to 8.516	0.695	0.488	0.685	1.459
SDS	0.499	0.140	0.266	0.222 to 0.775	3.569	**<0.001**	0.657	1.522
BAI	0.022	0.131	0.012	−0.237 to 0.280	0.165	0.869	0.703	1.423
GLTEQ	−0.162	0.072	−0.151	−0.305 to −0.019	−2.239	**0.027**	0.807	1.239
TSK	0.106	0.172	0.042	−0.235 to 0.447	0.616	0.539	0.780	1.282

Abbreviations: B = unstandardized regression coefficient; SE = standard error; β = standardized regression coefficient; CI = confidence interval; VIF = variance inflation factor; EDSS = Expanded Disability Status Scale; SDS = Zung Self-Rating Depression Scale; BAI = Beck Anxiety Inventory; GLTEQ = Godin Leisure-Time Exercise Questionnaire; TSK = Tampa Scale for Kinesiophobia; SF-36 BP = SF-36 Bodily Pain score; RRMS = relapsing–remitting multiple sclerosis; PPMS = primary progressive multiple sclerosis; SPMS = secondary progressive multiple sclerosis. Phenotype coefficients are adjusted differences from RRMS. ΔR^2^ denotes the joint contribution of all variables added in the specified block. All models used the same 148 participants. Changes in R^2^ were calculated from unrounded values.

## Data Availability

The data supporting the findings of this study are available from the corresponding author upon reasonable request. The data are not publicly available due to privacy and ethical restrictions related to participant confidentiality.

## Abbreviations

The following abbreviations are used in this manuscript:

BAI	Beck Anxiety Inventory
CI	Confidence Interval
EDSS	Expanded Disability Status Scale
GLTEQ	Godin Leisure-Time Exercise Questionnaire
IQR	Interquartile Range
MFIS	Modified Fatigue Impact Scale
MMSE	Mini-Mental State Examination
MS	Multiple Sclerosis
Q1–Q3	First to Third Quartile
SD	Standard Deviation
SDS	Zung Self-Rating Depression Scale
TSK	Tampa Scale for Kinesiophobia
VIF	Variance Inflation Factor
